# Trends in Staging, Treatment, and Survival in Colorectal Cancer Between 1990 and 2014 in the Rotterdam Study

**DOI:** 10.3389/fonc.2022.849951

**Published:** 2022-02-16

**Authors:** Birgit D. A. Lavrijssen, Rikje Ruiter, Jesse Fest, Mohammad A. Ikram, Bruno H. Stricker, Casper H. J. van Eijck

**Affiliations:** ^1^Department of Surgery, Erasmus Medical Centre, Rotterdam, Netherlands; ^2^Department of Epidemiology, Erasmus Medical Centre, Rotterdam, Netherlands; ^3^Department of Internal Medicine, Maasstad hospital, Rotterdam, Netherlands; ^4^Department of Surgery, Groene Hart Hospital, Gouda, Netherlands

**Keywords:** colorectal cancer, trends, tumor staging, treatment, survival

## Abstract

**Background:**

This study aims to assess trends in patient-related factors and treatment strategies in Dutch colorectal cancer (CRC) patients and their effect on survival.

**Methods:**

Data were obtained from the Rotterdam study, an ongoing population-based study of individuals aged ≥45 years. Between 1990 and 2014, incident, pathology-confirmed CRC cases were divided into two groups based on date of diagnosis (either before or after January 1, 2003). Patient characteristics, initial treatment, and date of mortality were collected. Analyses were performed using Kaplan–Meier and Cox proportional hazard models.

**Results:**

Of 14,928 individuals, 272 developed colon cancer and 124 rectal cancer. Median follow-up was 13.2 years. Patients diagnosed after January 1, 2003 were treated chemotherapeutically more often than those diagnosed prior to this date in colon cancer (28.6% vs. 9.1%, *p* = 0.02) and treated more often with chemotherapy (38.6% vs. 12.3%, *p* = 0.02) and radiotherapy (41.3% vs. 10.2%, *p* *=* 0.001) in rectal cancer. Overall survival, adjusted for patient, tumor characteristics, and treatment, improved in rectal cancer (HR, 0.31; 95% CI, 0.13–0.74) but remained stable in colon cancer (HR, 1.28; 95% CI, 0.84–1.95).

**Conclusion:**

Chemotherapeutic agents and radiotherapy are increasingly used in CRC patients. Survival in rectal cancer improved, whereas in colon cancer this was not observed.

## Introduction

With an estimated 1.8 million new cases and 881,000 deaths worldwide in 2018, colorectal cancer (CRC) is the third most commonly diagnosed malignancy and the second leading cause in cancer-related death (1). Both incidence and mortality are growing rapidly worldwide and are expected to increase to up to 2.2 million new cases and 1.1 million deaths by 2030 (2). Reasons for this increase are multifactorial and include aging, changing diet, and growth of the population, as well as changes in the prevalence and distribution of the main risk factors for colorectal cancer, several of which are associated with socioeconomic development (1, 3, 4).

These risk factors for CRC comprise both endogenous factors, such as (inherited) genetic predisposition, and exogenous factors such as lifestyle and environmental exposure (5). The influence of environmental factors on the incidence of CRC has been investigated extensively. Multiple studies have shown a positive association between both CRC incidence and mortality and smoking, alcohol consumption, obesity, and diabetes mellitus (DM) (6–15). In line with global trends in incidence and mortality rates in well-developed countries over the past few decades, incidence rates in the Netherlands increased from nearly 7,000 new colorectal cancer cases in 1990 to more than 15,000 new cases in 2014, while mortality rates in the same time period have decreased (16–18). After implementation of the national screening programme in 2014, incidence rates increased a little further to almost 16,000 new cases in 2015. Thereafter, a decrease was noted to nearly 14,000 new cases in 2018, with a further decrease expected to approximately 12,500 new cases in 2019 (18). Previous Dutch cohort studies, with data obtained from national registries, suggested that the decrease in mortality might be attributed to improvements in both early detection and treatment (2, 19–22). However, since they were unable to adjust for potential confounders, the influence of patient characteristics such as socioeconomic status (SES), presence of overweight, alcohol usage, smoking status, or presence of comorbidity on survival of CRC remains unknown.

Therefore, the aim of the present study was to describe changes in treatment and its effect on survival in patients with CRC in the Netherlands between 1990 and 2014. The secondary aim was to assess which patient-related and tumor-related factors were associated with survival.

## Materials and Methods

### Study Setting

Data were obtained from the Rotterdam study, a population-based prospective cohort study in Ommoord, a suburb in the city of Rotterdam in the Netherlands. The objectives and design of the Rotterdam study have been described extensively earlier (23, 24). Briefly, inhabitants aged 55 years and older were invited to participate in 1989. In total, 10,215 individuals were invited of whom 7,983 entered the study (78%). Between 2000 and 2001, a second cohort of 3,011 persons (67% response) aged 55 years and over was enrolled. A third cohort of 3,932 subjects of 45 years and older was enrolled in 2006 (65% response), leading to a total study population of 14,926 individuals, aged 45 years and over by the end of 2008. At baseline, participants underwent a home interview, as well as an extensive set of examinations at a research facility in the center of the district. These examinations included both a physical examination, imaging of the heart, blood vessels, eyes, skeleton and later brain, as well as collecting biospecimen to enable molecular and genetic analyses. Subsequent follow-up visits took place every 3–6 years.

### Case Identification

All incident CRC cases diagnosed between January 1, 1990 and January 1, 2015 in the Rotterdam study were included in this follow-up. Cancer cases were identified either through (1) follow-up of medical records of general practitioners, (2) discharge diagnosis registered by the Dutch Hospital Data (LMR) which captures discharge diagnoses for all nationwide hospital admissions, (3) pathology reports obtained through linkage with the nationwide network and registry of histo- and cytopathology in the Netherlands (PALGA), or (4) linkage with the National Cancer Registration (NKR), which captures additional information on tumor characteristics and initial treatment. Medical records, discharge letters, and pathology reports were revised by two medical doctors to obtain patient, tumor, and treatment characteristics. Patient characteristics included date of birth, medical history, and several modifiable lifestyle factors, tumor characteristics comprised tumor type and differentiation grade, while treatment characteristics comprehended date, type and aim of a surgical procedure, indication and treatment with chemotherapy and indication and aim of radiotherapy, among others. All obtained patient, tumor and treatment characteristics are described in **Tables 1**–**3**. The level of certainty was diagnosed as either certain (histopathologically confirmed), probable (clinical diagnosis based on radiological examination and/or biomarkers) or probable (clinical diagnosis by physical examination). Only certain cases were included in analyses. In case of discrepancy, consensus was sought through consultation of a specialist in internal medicine. Cancer follow-up was complete until January 1, 2015 and information on vital status until January 1, 2020. Patients with a prior history of cancer (excluding non-melanoma skin cancer), as well as cases discovered at autopsy were excluded from the analyses. Patients with tumors of the appendix or anus were not considered cases. In the case of metachronous primary tumors, only the first diagnosed CRC was included. In the case of synchronous CRC, the tumor with the highest stage in the tumor, node, and metastases (TNM) staging system was included. Follow-up time was defined as the period between the date of diagnosis of CRC and date of death, censoring, or end of study period (January 1, 2020), whichever came first.

### Tumor Classification

Classification of cases was according to the International Classification of Diseases and Related Health Problems, 10th revision (ICD-10), and patients were stratified by tumor localization in either colon (C18) or rectum (rectosigmoid and rectum, C19 and C20). The date of diagnosis was registered as the date of the pathology report or the date of hospital admission if no pathology report was available yet. The TNM classification was coded according to the edition valid at time of diagnosis (1990–1996 4th edition, 1997–2002 5th edition, 2003–2009 6th edition, 2010–2014 7th edition).

Based on date of diagnosis, our study cohort was then divided into two groups (either 1990–2002 or 2003–2014), as from the 6th edition onwards, tumor stage II was subdivided as IIA and IIB (on the basis whether the tumor was T3 or T4, respectively), and stage III was subdivided into IIIA (T1-2N1M0), IIIB (T3-4N1M0), or IIIC (any TN2M0), see **Supplementary Table 1**. Subsequently, disease stages in those diagnosed after January 1, 2003 were transformed to the corresponding disease stage in those diagnosed before January 1, 2003. Information on vital status was obtained from the national database of deceased persons of the Central Bureau for Genealogy. Follow-up was complete until January 1, 2020.

### Covariables

The following covariables were considered potential determinants of survival: age, sex, body-mass index (BMI), smoking status (current, former, never), use of alcohol (yes/no), SES based on level of education (primary/lower vocational/intermediate vocational/higher vocational or university), and presence of DM. DM at date of diagnosis was based on a fasting plasma glucose level ≥7.0 mmol/L or nonfasting plasma glucose level ≥11.6 mmol/L or use of glucose-lowering drugs. Treatment was assessed through resection of the primary tumor yes/no, type of operation (open procedure vs. laparoscopic procedure), chemotherapy (yes/no and neoadjuvant/adjuvant/palliative), and radiotherapy (yes/no and neoadjuvant/adjuvant/palliative).

### Statistical Analysis

Categorical variables were expressed in frequencies and percentages, while continuous variables were expressed in means and standard deviation (SD). As mentioned before, participants were divided into two groups based on date of diagnosis (either before or after January 1, 2003) to assess trends in diagnostics and treatment within our cohort. Fisher’s exact test and Chi-squared test were used for comparisons of categorical variables, such as tumor characteristics, and Student’s *t*-test and Mann-Whitney *U* test for continuous parametric and nonparametric variables, respectively.

Survival curves were plotted using the Kaplan-Meier method and compared using the Log-Rank test. Survival percentages in our cohort were compared with the nationwide survival percentages published by the NKR using the Z-score test.

Cox proportional hazard regression analyses was used to assess risk of mortality. Proportionality was tested by constructing log-log plots for each variable separately. Variables in which the Cox proportional hazards assumptions did not hold, were added as time-dependent variables to the model to estimate hazard ratios (HR) and 95% confidence intervals (CI) for overall mortality. All multivariable models were adjusted for age at diagnosis, sex, and year of diagnosis (dichotomous: 1990–2002, 2003–2014). Additionally, analyses were adjusted for patient characteristics, tumor characteristics and treatment strategy. All analyses were performed for colon and rectal cancer separately.

As a sensitivity analysis, we also calculated overall conditional survival for both colon and rectal cancer in the total study cohort and per age group and disease stage separately. Conditional survival is the probability of surviving another *t* years, given that a subject has survived *x* years after diagnosis and can be calculated by *S* (*x* + *t*)/*S*(*x*) (25).

*p-*values <0.05 were considered statistically significant. Analyses were performed using SPSS Statistics for Windows (version 25.0, IBM Corp, Armonk, NY) and R version 3.6.1 (https://www.r-project.org/).

## Results

In total, 396 individuals with an incident diagnosis of a histopathological proven primary CRC were divided into a colon cancer cohort (*n* = 272, with a tumor in either caecum, ascending colon, hepatic flexure, transverse colon, splenic flexure, descending colon, and sigmoid colon) and a rectal cancer cohort (*n* = 124, with a tumor in either rectosigmoid or rectum, see **Supplementary Figure S1**). As previously described, analyses were split for cases with a date of diagnosis between January 1, 1990 and December 31, 2002 and cases with a date of diagnosis between January 1, 2003 and December 31, 2014 (26).

### Colon Cancer

In our colon cancer cohort, patients diagnosed after January 1, 2003 were more often former smokers (*p* = 0.05) and current alcohol users (*p* = 0.001) than those diagnosed before 2003. No clear significant differences in age, sex, BMI, presence of DM, and socioeconomic status were observed between the different study groups (see **Table 1**).

**Table 1:** Patient characteristics.

	Colon cancer cohort			Rectal cancer cohort		
	1990–2002	2003–2014	*p*-value	1990–2002	2003–2014	*p*-value
	*N* = 124	*N* = 148		*N* = 49	*N* = 75	
Age (years)						
Mean (SD)	76.0 (8.1)	76.5 (8.4)	0.63	76.2 (7.8)	73.9 (8.3)	0.13
Age category						
<75	52 (42.0%)	55 (37.2%)	0.72	24 (49.0%)	43 (57.3%)	0.66
75–85	57 (46.0%)	74 (50.0%)		18 (36.7%)	23 (30.7%)	
>85	15 (12.1%)	19 (12.8%)		7 (14.3%)	9 (12.0%)	
Sex						
Male	53 (42.7%)	73 (49.3%)	0.28	22 (44.9%)	37 (49.3%)	0.63
Female	71 (57.3%)	75 (50.7%)		27 (55.1%)	38 (50.7%)	
BMI (kg/m^2^)						
Mean (SD)	26.1 (3.5)	26.9 (3.3)	0.07	26.4 (4.0)	27.5 (3.9)	0.17
BMI (kg/m^2^)						
<25.00	46 (39.3%)	40 (28.6%)	0.06	18 (42.9%)	19 (25.7%)	0.15
25.0–29.99	58 (49.6%)	71 (50.7%)		16 (38.1%)	39 (52.7%)	
>30	13 (11.1%)	29 (20.7%)		8 (19.0%)	16 (21.6%)	
Prevalent DM						
No	99 (83.9%)	118 (80.8%)	0.52	37 (82.2%)	59 (79.7%)	0.74
Yes	19 (16.1%)	28 (19.2%)		8 (17.8%)	15 (20.3%)	
Alcohol						
Never	17 (13.7%)	10 (6.8%)	0.001	8 (16.3%)	4 (5.3%)	0.005
Former	6 (4.8%)	5 (3.4%)		2 (4.1%)	6 (8.0%)	
Current	90 (72.6%)	132 (89.2%)		31 (63.3%)	63 (84.0%)	
Smoking						
Never	45 (37.8%)	41 (28.5%)	0.05	19 (39.6%)	18 (25.7%)	0.25
Former	52 (43.7%)	85 (59.0%)		21 (43.8%)	35 (50.0%)	
Current	22 (18.5%)	18 (12.5%)		8 (16.7%)	17 (24.3%)	
SES						
Very low	32 (26.2%)	23 (15.5%)	0.15	10 (20.8%)	8 (10.8%)	0.14
Low	48 (39.3%)	66 (44.6%)		23 (47.9%)	31 (41.9%)	
Medium	31 (25.4%)	39 (26.4%)		11 (22.9%)	19 (25.7%)	
High	11 (9.0%)	20 (13.5%)		4 (8.3%)	16 (21.6%)	
Mortality						
No	7 (5.6%)	40 (27.0%)	<0.001	7 (14.3%)	26 (34.7%)	0.01
Yes	117 (94.4%)	108 (73.0%)		42 (85.7%)	49 (65.3%)	

Missing colon cancer cohort: BMI, n = 16 (5.9%); prevalent DM, n = 8 (2.9%); alcohol status, n = 12 (4.4%); smoking, n = 9 (3.3%); SES, n = 2 (0.7%).

Missing rectal cancer cohort: BMI, n = 8 (6.5%); prevalent DM, n = 5 (4.0%); alcohol status, n = 8 (6.5%); smoking, n = 6 (4.8%); SES, n = 2 (1.6%).

T-stage, N-stage, and disease stage, as well as other tumor characteristics, such as morphology, differentiation grade, and tumor subsite, did not change significantly over time between both groups. However, M-stage was more often known in those diagnosed after 2003 (*p* < 0.001, see **Table 2**). However radiological imaging was performed more often to obtain information on possible lymph node metastases (p <0.001, see **Table 2**).

**Table 2:** Tumor characteristics.

	Colon cancer cohort			Rectal cancer cohort		
	1990–2002	2003–2014	*p*-value	1990–2002*N* = 49	2003–2014*N* = 75	*p*-value
	*N* = 124	*N* = 148				
T-stage						
T0	NA	NA	0.65	0 (0.0%)	1 (1.3%)	0.88
T1	14 (11.3%)	12 (8.1%)		6 (12.2%)	9 (12.0%)	
T2	23 (18.5%)	22 (14.9%)		9 (18.4%)	19 (25.3%)	
T3	65 (52.4%)	79 (53.4%)		25 (51.0%)	34 (45.3%)	
T4	12 (9.7%)	18 (12.2%)		3 (6.1%)	5 (6.7%)	
Missing	10 (8.1%)	17 (11.5%)		6 (12.2%)	7 (9.3%)	
N-stage						
N0	65 (62.4%)	78 (52.7%)	0.67	21 (42.9%)	32 (42.7%)	0.05
N1	21 (16.9%)	27 (18.2%)		17 (34.7%)	16 (21.3%)	
N2	10 (8.1%)	18 (12.2%)		3 (6.1%)	16 (21.3%)	
N3	1 (0.8%)	0 (0.0%)		0 (0.0%)	0 (0.0%)	
Missing	28 (22.6%)	25 (16.9%)		8 (16.3%)	11 (14.3%)	
M-stage						
M0	41 (33.1%)	81 (54.7%)	<0.001	18 (36.7%)	41 (54.7%)	0.004
M1	26 (21.0%)	36 (24.3%)		7 (14.3%)	20 (26.7%)	
Missing	57 (46.0%)	31 (21.0%)		24 (48.9%)	14 (18.7%)	
Stage						
I	28 (22.6%)	32 (21.6%)	0.97	12 (24.5%)	22 (29.3%)	0.16
II	42 (33.9%)	48 (32.4%)		11 (22.4%)	12 (16.0%)	
III	25 (20.2%)	28 (18.9%)		15 (30.6%)	20 (26.7%)	
IV	26 (21.0%)	35 (23.6%)		7 (14.3%)	20 (26.7%)	
Morphology						
adenocarcinoma	118 (95.2%)	137 (92.6%)	0.56	43 (87.8%)	65 (86.7%)	0.50
NET = neuroendocrine tumor	1 (0.8%)	1 (0.7%)		0 (0.0%)	0 (0.0%)	
Squamous	1 (0.8%)	0 (0.0%)		6 (12.2%)	8 (10.7%)	
Signet cell	2 (1.6%)	4 (2.7%)		0 (0.0%)	2 (2.7%)	
Differentiation grade						
Well	13 (11.0%)	5 (4.0%)	0.16	7 (15.2%)	2 (3.3%)	0.07
Moderate	84 (71.2%)	95 (76.6%)		31 (67.4%)	50 (82.0%)	
Poorly	20 (16.9%)	21 (16.9%)		8 (17.4%)	9 (14.8%)	
Undiff. = Undifferentiated	1 (0.8%)	3 (2.4%)		0 (0.0%)	0 (0.0%)	
Tumor location						
Left	116 (67.4%)	137 (61.7%)	0.46	NA	NA	0.02
Transverse	6 (3.5%)	11 (5.0%)		NA	NA	
Right	50 (29.1%)	74 (33.3%)		NA	NA	
Rectum	NA	NA		33 (67.3%)	64 (85.3%)	
Rectosigmoid	NA	NA		16 (32.7%)	11 (14.7%)	
Relapse						
Local	5 (4.0%)	5 (3.4%)	0.79	0	0	0.82
Local and distant	5 (4.0%)	5 (3.4%)		2 (4.3%)	4 (5.3%)	
Distant	16 (12.9%)	14 (9.5%)		6 (12.8%)	7 (9.3%)	
None	98 (79.0%)	124 (83.8%)		39 (83.0%)	64 (85.3%)	

Missing colon cancer cohort: disease stage, n = 8 (2.9%); morphology, n = 8 (2.9%).

Missing rectal cancer cohort: disease stage, n = 5 (4.0%).

NA, Not Applicable.

With regard to treatment, the proportion of patients who underwent a resection of the primary tumor remained stable, whereas the proportion of laparoscopic procedures increased statistically significantly from 5% in patients diagnosed before 2003 to over 30% (*p* < 0.001) in those diagnosed after January 1, 2003 (**Table 3**). Furthermore, patients diagnosed after January 1, 2003 were treated with chemotherapeutic agents more often (*p* = 0.02), either adjuvant with a curative intention in stage III patients (28.6% vs. 9.1% respectively, *p* = 0.06), or palliative in case of stage IV disease (58.8% vs. 28.0% respectively, *p* = 0.02).

**Table 3:** Treatment characteristics.

	Colon cancer cohort			Rectal cancer cohort		
	1990–2002	2003–2014	*p*-value	1990–2002	2003–2014	*p*-value
	*N* = 124	*N* = 148		*N* = 49	*N* = 75	
Resection primary tumor						
Yes	110 (90.2%)	124 (83.8%)	0.13	43 (89.6%)	60 (80.0%)	0.16
No	12 (9.8%)	24 (16.2%)		5 (10.4%)	15 (20.0%)	
Open vs. laparoscopic						
Open	112 (93.3%)	91 (67.9%)	<0.001	41 (89.1%)	44 (68.8%)	0.04
Laparoscopic	6 (5.0%)	41 (30.6%)		4 (8.7%)	17 (26.6%)	
Treatment intention						
Curative	94 (78.3%)	115 (85.8%)	0.17	38 (82.6%)	55 (85.9%)	0.24
Palliative	22 (18.3%)	18 (13.4%)		6 (13.0%)	9 (14.1%)	
Chemotherapy						
None	107 (86.3%)	112 (75.7%)	0.02	41 (83.7%)	45 (60.0%)	0.01
Neoadjuvant	NA	3 (2.0%)		0 (0.0%)	10 (13.3%)	
Adjuvant	2 (1.6%)	11 (7.4%)		2 (4.1%)	10 (13.3%)	
Palliative	11 (8.9%)	21 (14.2%)		4 (8.2%)	9 (12.0%)	
Radiotherapy						
None	123 (99.2%)	143 (96.6%)	0.15	42 (85.7%)	41 (54.7%)	0.001
Neoadjuvant	NA	NA		5 (10.2%)	30 (40.0%)	
Adjuvant	NA	NA		0 (0.0%)	1 (1.3%)	
Palliative	1 (0.8%)	5 (3.4%)		0 (0.0%)	3 (4.0%)	

Missing colon cancer cohort: open vs. laparoscopic, n = 4 (1.5%); treatment intention, n = 5 (1.8%); chemotherapy, n = 5 (1.8%).

Missing rectal cancer cohort: open vs. laparoscopic, n = 4 (3.2%); treatment intention, n = 2 (1.6%), chemotherapy, n = 3 (2.4%).

NA, Not Applicable.

**Figure 1:**
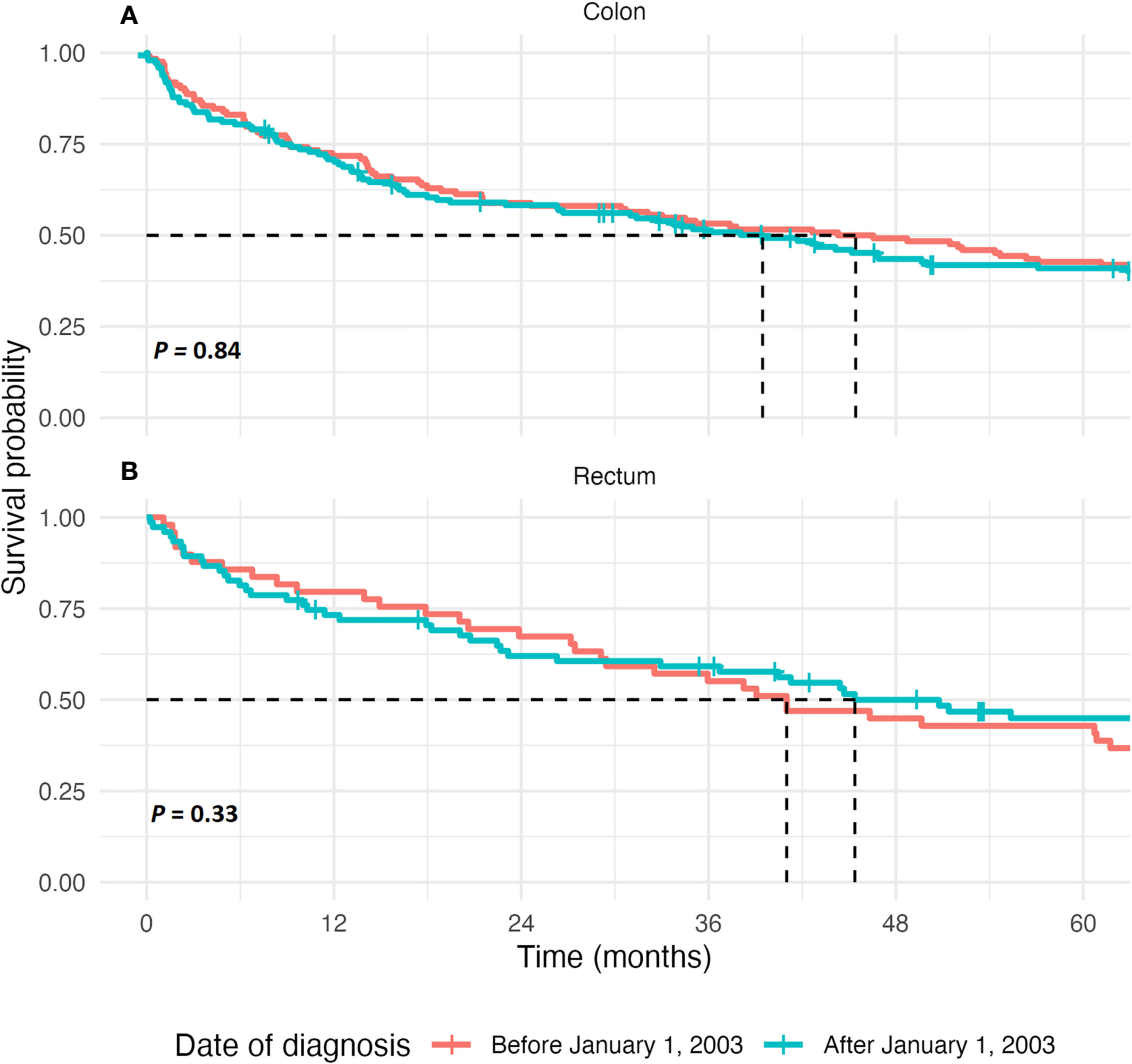
Kaplan-Meier estimate of survival in colon cancer patients and rectal cancer patients. **(A)** The 5-year overall survival, in months, after diagnosis for patients of all tumor stages combined in colon cancer patients. The curves are compared using the Log-Rank test. **(B)** The 5-year overall survival, displayed in months, after diagnosis for patients of all tumor stages combined in rectal cancer patients. The curves are compared using the Log-Rank test.

Median overall survival in our cohort did not improve significantly over time in all stages combined (44.3 months in those diagnosed before and 45.2 months in those diagnosed after January 1, 2003, respectively, *p* = 0.84), nor when only stages I, II, and III were combined (77.8 vs. 82.6 months, respectively, *p* = 0.83), as is shown in **Figure 1** and **Supplementary Tables S2, S4**. Risk of mortality did not differ between both groups when adjusted for patient-related factors, tumor characteristics, and treatment strategy (HR, 1.28; 95% CI, 0.84–1.95, see **Supplementary Table S5**).

The 5-year survival rates ranged from 77% in stage I colon cancer cases to only 2% in stage IV colon cancer cases. In our cohort, combined 1-year survival did not improve and was stable around 72%; however, 5-year survival for all CRC cases increased from 42.7% to 44.6%, albeit nonstatistically significant (*p* = 0.76).

The nationwide absolute overall 1-year survival percentages in colon cancer patients in these time periods increased from 71.1% to 76.1% whereas the 5-year survival percentage improved from 43.5% to 51.3%, but this did not differ significantly from our observed percentages (*p* = 0.89).

### Rectal Cancer

In our rectal cancer cases, no significant differences in BMI, presence of DM, smoking status, and SES were found in those diagnosed before January 1, 2003 compared with those diagnosed after January 1, 2003. However, cases with a diagnosis after January 1, 2003 were more often current alcohol users (*p* = 0.005, see **Table 1**). No significant differences were found in disease stage and other tumor characteristics, while information on possible lymph node or distant metastasis was more often available (*p* = 0.004, see **Table 2**). Although resection rates remained stable over time, the procedure was significantly more often performed laparoscopically in those diagnosed after January 1, 2003 compared with those diagnosed prior to this date (26.6% and 8.7%, respectively, *p* = 0.04; see **Table 3**).

In patients diagnosed before the January 1, 2003, 71.4% of all stage IV patients underwent a resection of the primary tumor, versus 35.0% diagnosed after this date (*p* = 0.09). Although statistically nonsignificantly different, stage IV patients diagnosed after January 1, 2003 were treated more often with chemotherapy, both neoadjuvant and adjuvant, while none of the stage IV patients diagnosed before 2003 were treated chemotherapeutically (15.8% vs. 0.0% respectively, *p* = 0.74).

Similar results were found with regard to radiotherapy. Of all stage IV subjects diagnosed after January 1, 2003, 20% underwent neoadjuvant radiotherapy, whereas none of the stage IV patients diagnosed before 2003 underwent radiotherapy, *p-*value 0.31. However, the number of cases in both groups is small (1990–2002, *n* = 7; 2003–2014, *n* = 20).

Median overall survival did not improve significantly over time in all stages combined (41.0 months in those diagnosed before and 55.4 months in those diagnosed after January 1, 2003, respectively, *p* = 0.33), nor when only stages I, II, and III were combined (60.7 vs. 100.7 months, respectively, *p* = 0.07), as is shown in **Figure 1**, **Supplementary Tables S3, S4** and **Supplementary Figure S2**.

When adjusted for patient, tumor characteristics, and treatment strategy, risk of mortality in subjects diagnosed after January 1, 2003 decreased by 69% (HR, 0.31; 95% CI, 0.13–0.74, see **Supplementary Table S5**).

In our cohort, the 5-year survival percentage of stage I rectal cancer cases was 67.6%, while only one of the stage IV rectal cancer cases (3.7%) was alive 5 years after diagnosis. The combined 1-year survival in our cohort decreased from 79.6% in those diagnosed before 2003 to 73.3% in subjects diagnosed after January 1, 2003, whereas an increase in 5-year survival was observed from 42.9% to 49.3%, respectively (*p* *=* 0.43 and *p* = 0.48, respectively). Nationwide, 1- and 5-year absolute overall survival in rectal cancer increased from 76.8% to 82.7% and from 44.9% to 55.9%, respectively, again not statistically different from our observed survival percentages (*p* = 0.85).

### Sensitivity Analysis

The 1-year conditional survival in colon cancer patients increased from 83% to 92% in the first 5 years after diagnosis and did not differ between both groups. The 5-year conditional survival ranged from 69% to 77% in the first 5 years after diagnosis in subjects diagnosed before January 1, 2003, whereas for those diagnosed after 2003, these percentages were 57% to 70%.

In rectal cancer patients, the 1-year conditional survival did not differ between both groups, remained relatively stable and ranged between 85% after 1 year and 95% 4 years after diagnosis. The 5-year conditional survival improved from 46% 1 year after diagnosis to 66.7% 5 years after diagnosis in subjects diagnosed between 1990 and 2003. In subjects diagnosed between 2003 and 2014, these percentages increased from 61.7% to 70.4%, respectively.

## Discussion

We observed a decreased risk of mortality in rectal cancer subjects diagnosed after January 1, 2003 compared with those diagnosed before this date, whereas in colon cancer subjects, no change over time was observed. Kaplan–Meier estimates of survival showed no differences in both rectal and colon cancer subjects. While resection rates remained stable and high, over time procedures were more often performed laparoscopically and both colon and rectal cancer patients were more often treated chemotherapeutically.

In diagnosing colorectal cancer cases, information on possible lymph node and distant metastases was obtained and reported significantly more often over time, illustrated by the decrease in the proportion of patients in whom information on lymph node metastasis (22.6% vs. 16.9% in the colon cancer cohort and 16.3% vs. 14.3% in our rectal cancer cohort) and distant metastasis (46.0% vs. 21.0% in colon cancer and 48.9% vs. 18.7% in rectal cancer, respectively) was not available, which is in line with a previous nationwide Dutch study (27). During our study period, advancements in imaging techniques, such as more widespread use of CT and MRI scanning, have emerged, leading to detection of smaller metastases, which would otherwise have remained undetected (28, 29). Nonetheless, no evidence was found that more recently diagnosed patients as a result were diagnosed at an earlier stage yet. In 2014, a nationwide screening program was implemented in the Netherlands. Studies conducted in the other parts of Western Europe and the United States observed a trend towards an earlier stage and less advanced disease at diagnosis, leading to a significant decrease in mortality rates (30–32). Nationwide mortality rates in the Netherlands have decreased modestly over the last decades and are, partly due to the screening program, expected to decrease further (27, 33). The number of cases from the national screening program included in our cohort was too low to address this question.

Our findings of stable resection rates over time in stages I–III patients were also demonstrated in a French population-based study, and also our findings with regard to changes in both detection and treatment in both colon and rectal cancer, are in line with a previous Dutch study (17, 34). As shown by multiple studies, the proportion of patients receiving adjuvant chemotherapy (both curative and palliative) increased, with the increase being steeper in stage III patients and smaller in older patients (aged >75 years) (17, 34). This is similar to our findings. In colon cancer, chemotherapy was mainly administered as adjuvant therapy, whereas rectal cancer cases mainly received chemotherapy as neoadjuvant treatment. In our cohort, none of the ten stage III patients aged >85 years received adjuvant treatment with either chemotherapy or radiotherapy. Previous, mainly retrospective, studies have shown conflicting results in benefits of adjuvant therapy in elderly patients, ranging from no benefit and increased toxicity to similar benefits from adjuvant chemotherapy in elderly patients as compared with younger patients, without a significant increase in toxicity (35). The Dutch Colorectal cancer guideline recommends an individual approach in elderly patients to carefully weigh harms against benefits, taking performance status and comorbidities into account instead of age.

Overall survival in our colon cancer cases did not improve over time. This might, at least partly, be explained by changes in diet and lifestyle over time, as subjects diagnosed after 2003 were more often overweight, (former) smokers and (current) alcohol users. Previous studies have shown an increased mortality (both overall mortality and cancer-specific mortality) for current and former smokers, alcohol users, and obese CRC patients (6–14). Another, and maybe more likely, explanation might be the age distribution of our study population. A previous Dutch cohort study found improved 5-year survival rates from 51% in 1975–1984 to 58% in 2000–2004 in colon cancer patients, and survival rates in rectal cancer patients improved from 44% to 59% in that period (17). In younger patients (aged <70 years), these survival rates increased throughout the whole study period, while in elderly patients, this increase was only seen up to 1995–1999 (17). In their study, over 30% of all patients were younger than 65 years old, while in our study almost 90% of all subjects were aged 65 years and over and almost 75% were aged >70 years.

Other studies reported relative survival and disease-specific survival, while we report absolute all-cause mortality numbers. We therefore obtained age-specific national data on absolute overall survival in both colon and rectal cancer. The proportion of younger patients (<75 years) in both our colon cancer and rectal cancer cohort were smaller than nationwide. This might be explained by the age limit in our cohort of 55 years and older for the first two included cohorts (1990-2005) and 45 years and over for the third cohort. Nationwide, the proportion of colon cancer patients aged <75 years remained stable around 60% (95% CI, 60–62), whereas in our cohort, these percentages were 41.9% (95% CI, 33–51) in those diagnosed before January 1, 2003 and 37.1% (95% CI, 29–45) in those diagnosed after January 1, 2003 (*p* < 0.001). In our rectal cancer cohort, 49.0% (95% CI, 35–63) of the patients diagnosed before January 1, 2003 and 57.3% (95% CI, 46–69) of those diagnosed after January 1, 2003 were aged <75 years. This was significantly smaller than nationwide, where this proportion remained stable around 70% (95% CI, 68–71; *p* = 0.005). As mentioned before, previous studies have shown that the improvement in survival was much more prominent in younger patients.

Our Cox proportional hazards model showed a lower risk of mortality in rectal cancer patients after adjustment for patient-related factors, tumor characteristics, and treatment strategy, suggesting a significant contribution in the improvement of survival. Resection rates in our cohort were consistently high over 80% in all stages in both colon and rectal cancer, while the proportion of laparoscopic procedures significantly increased over time. Median overall survival in laparoscopically operated patients was significantly higher than those who underwent an open procedure when stratified for diagnose before or after January 1, 2003 (*p* = 0.008 in colon cancer patients and *p* = 0.04 in rectal cancer patients, data not shown). This, however, can be explained by the stage distribution, as laparoscopically operated patients were more often diagnosed with stage I or II cancer (*p* = 0.02 in our colon cancer cohort and *p* = 0.001 in our rectal cancer cohort, data not shown). The European Multicenter Colon Cancer Laparoscopic or Open Resection (COLOR) trial, a large, prospective, randomized controlled trial conducted in 29 participating hospitals in Europe, demonstrated no differences in both disease-free and overall survival at 3 and 10 years follow-up between laparoscopic and open surgery for colon cancer with tumor stages being equally distributed among both types of procedures (36, 37). Similar results were found in rectal cancer patients (38). In addition, treatment of rectal cancer has changed drastically during our study, with implementation of the total mesorectal excision (TME) approach and increased neoadjuvant administration of chemo- and radiotherapy, leading to an increased survival as shown in previous studies (22, 39, 40). In metastasized patients, a shift was observed from resection of the primary tumor towards systemic therapy with chemotherapy. In our cohort however, no improvement in absolute overall survival or conditional survival was observed.

With its long follow-up and detailed information on important risk factors and comorbidities, a large population-based cohort study, such as the Rotterdam study, enables evaluation of changes in patient characteristics, diagnostics, and implemented care. However, an important limitation of our study is the relatively small number of included cases, causing overfitting in some of our multivariate Cox proportional hazard models.

In conclusion, between 1990 and 2014, resections in colorectal cancer patients were performed laparoscopically more often and patients were treated chemotherapeutically more frequently. Absolute overall survival in rectal cancer patients improved, while absolute overall survival in colon cancer patients remained stable. Lack of improvement of survival in our cohort may be explained by the large proportion of elderly, compared with the general population and the difference in survival benefit of treatment between younger and elderly patients.

## Data Availability Statement

Because of restrictions based on privacy regulations and informed consent of the participants, data cannot be made freely available in a public repository. Requests to access these datasets should be directed to the management team of the Rotterdam study (datamanagement.ergo@erasmusmc.nl), which has a protocol for approving data requests.

## Ethics Statement

The studies involving human participants were reviewed and approved by the Medical Ethics Committee of the Erasmus MC (registration number MEC 02.1015) and by the Dutch Ministry of Health, Welfare and Sport (Population Screening Act WBO, license number 1071272-159521-PG). The Rotterdam Study Personal Registration Data collection is filed with the Erasmus MC Data Protection Officer under registration number EMC1712001. The Rotterdam study has been entered into the Netherlands National Trial Register (NTR; www.trialregister.nl) and into the WHO International Clinical Trials Registry Platform (ICTRP; https://apps.who.int/trialsearch/) under shared catalogue number NL6645/NTR6831. All participants provided written informed consent to participate in the study and to have their information obtained from treating physicians. The patients/participants provided their written informed consent to participate in this study.

## Author Contributions

Conception and study design: BL, JF, TR, BS, and CE. Data collection: all. Data analysis: BL, JF, and TR. Supervision: TR, BS, and CE. Writing original draft: BL and TR. Editing and final approval: all. All authors listed have made a substantial, direct, and intellectual contribution to the work and approved it for publication.

## Conflict of Interest

The authors declare that the research was conducted in the absence of any commercial or financial relationships that could be construed as a potential conflict of interest.

## Publisher’s Note

All claims expressed in this article are solely those of the authors and do not necessarily represent those of their affiliated organizations, or those of the publisher, the editors and the reviewers. Any product that may be evaluated in this article, or claim that may be made by its manufacturer, is not guaranteed or endorsed by the publisher.

## References

[1] Bray F Ferlay J Soerjomataram I Siegel RL Torre LA Jemal A. Global Cancer Statistics 2018: GLOBOCAN Estimates of Incidence and Mortality Worldwide for 36 Cancers in 185 Countries. CA Cancer J Clin (2018) 68(6):394–424. doi: 10.3322/caac.21492 30207593

[2] Arnold M Sierra MS Laversanne M Soerjomataram I Jemal A Bray F. Global Patterns and Trends in Colorectal Cancer Incidence and Mortality. Gut (2017) 66(4):683–91. doi: 10.1136/gutjnl-2015-310912 26818619

[3] Gersten O Wilmoth JR. The Cancer Transition in Japan Since 1951. Demogr Res (2002) 7:271–306. doi: 10.4054/DemRes.2002.7.5

[4] Omran AR. The Epidemiologic Transition: A Theory of the Epidemiology of Population Change. Milbank Memorial Fund Q (1971) 49(4):509–38. doi: 10.2307/3349375 5155251

[5] Aran V Victorino AP Thuler LC Ferreira CG. Colorectal Cancer: Epidemiology, Disease Mechanisms and Interventions to Reduce Onset and Mortality. Clin Colorectal Cancer (2016) 15(3):195–203. doi: 10.1016/j.clcc.2016.02.008 26964802

[6] Botteri E Iodice S Bagnardi V Raimondi S Lowenfels AB Maisonneuve P. Smoking and Colorectal Cancer: A Meta-Analysis. JAMA (2008) 300(23):2765–78. doi: 10.1001/jama.2008.839 19088354

[7] Liang PS Chen TY Giovannucci E. Cigarette Smoking and Colorectal Cancer Incidence and Mortality: Systematic Review and Meta-Analysis. Int J Cancer (2009) 124(10):2406–15. doi: 10.1002/ijc.24191 19142968

[8] Jayasekara H English DR Haydon A Hodge AM Lynch BM Rosty C. Associations of Alcohol Intake, Smoking, Physical Activity and Obesity With Survival Following Colorectal Cancer Diagnosis by Stage, Anatomic Site and Tumor Molecular Subtype. Int J Cancer (2018) 142(2):238–50. doi: 10.1002/ijc.31049 28921583

[9] Walter V Jansen L Hoffmeister M Ulrich A Chang-Claude J Brenner H. Smoking and Survival of Colorectal Cancer Patients: Population-Based Study From Germany. Int J Cancer (2015) 137(6):1433–45. doi: 10.1002/ijc.29511 25758762

[10] Fedirko V Tramacere I Bagnardi V Rota M Scotti L Islami F. Alcohol Drinking and Colorectal Cancer Risk: An Overall and Dose-Response Meta-Analysis of Published Studies. Ann Oncol (2011) 22(9):1958–72. doi: 10.1093/annonc/mdq653 21307158

[11] Huxley RR Ansary-Moghaddam A Clifton P Czernichow S Parr CL Woodward M. The Impact of Dietary and Lifestyle Risk Factors on Risk of Colorectal Cancer: A Quantitative Overview of the Epidemiological Evidence. Int J Cancer (2009) 125(1):171–80. doi: 10.1002/ijc.24343 19350627

[12] Ma Y Yang Y Wang F Zhang P Shi C Zou Y. Obesity and Risk of Colorectal Cancer: A Systematic Review of Prospective Studies. PloS One (2013) 8(1):e53916. doi: 10.1371/journal.pone.0053916 23349764 PMC3547959

[13] Larsson SC Wolk A. Obesity and Colon and Rectal Cancer Risk: A Meta-Analysis of Prospective Studies. Am J Clin Nutr (2007) 86(3):556–65. doi: 10.1093/ajcn/86.3.556 17823417

[14] Bardou M Barkun AN Martel M. Obesity and Colorectal Cancer. Gut (2013) 62(6):933–47. doi: 10.1136/gutjnl-2013-304701 23481261

[15] Jiang Y Ben Q Shen H Lu W Zhang Y Zhu J. Diabetes Mellitus and Incidence and Mortality of Colorectal Cancer: A Systematic Review and Meta-Analysis of Cohort Studies. Eur J Epidemiol (2011) 26(11):863–76. doi: 10.1007/s10654-011-9617-y 21938478

[16] Brouwer NPM Bos A Lemmens V Tanis PJ Hugen N Nagtegaal ID. An Overview of 25 Years of Incidence, Treatment and Outcome of Colorectal Cancer Patients. Int J Cancer (2018) 143(11):2758–66. doi: 10.1002/ijc.31785 PMC6282554 30095162

[17] Lemmens V van Steenbergen L Janssen-Heijnen M Martijn H Rutten H Coebergh JW. Trends in Colorectal Cancer in the South of the Netherlands 1975-2007: Rectal Cancer Survival Levels With Colon Cancer Survival. Acta Oncol (2010) 49(6):784–96. doi: 10.3109/02841861003733713 20429731

[18] Nederlandse Kankerregistratie. Utrecht: IKNL, NCCO (2020). Available at: https://iknl.nl/nkr-cijfers. (Accessed July 29, 2020)

[19] Sandler RS. Epidemiology and Risk Factors for Colorectal Cancer. Gastroenterol Clin North Am (1996) 25(4):717–35. doi: 10.1016/S0889-8553(05)70271-5 8960889

[20] Murphy CC Harlan LC Lund JL Lynch CF Geiger AM. Patterns of Colorectal Cancer Care in the United States: 1990-2010. J Natl Cancer Inst (2015) 107(10):djv198. doi: 10.1093/jnci/djv198 26206950 PMC4840367

[21] Martijn H Voogd AC van de Poll-Franse LV Repelaer van Driel OJ Rutten HJ Coebergh JW. Improved Survival of Patients With Rectal Cancer Since 1980: A Population-Based Study. Eur J Cancer (2003) 39(14):2073–9. doi: 10.1016/S0959-8049(03)00493-3 12957462

[22] den Dulk M Krijnen P Marijnen C Rutten H van de Poll Franse L Putter H. Improved Overall Survival for Patients With Rectal Cancer Since 1990: The Effects of TME Surgery and Pre-Operative Radiotherapy. Eur J Cancer (2008) 44:1710–6. doi: 10.1016/j.ejca.2008.05.004 18573654

[23] Iskram MA Brusselle G Ghanbari M Goedegebure A Ikram MK Kavousi M. Objectives, Design and Main Findings Until 2020 From the Rotterdam Study. Eur J Epidemiol (2020) 35(5):483–517. doi: 10.1007/s10654-020-00640-5 32367290 PMC7250962

[24] Hofman A Grobbee DE de Jong PT van den Ouweland FA. Determinants of Disease and Disability in the Elderly: The Rotterdam Elderly Study. Eur J Epidemiol (1991) 7(4):403–22. doi: 10.1007/BF00145007 1833235

[25] Belot A Ndiaye A Luque-Fernandez MA Kipourou DK Maringe C Rubio FJ. Summarizing and Communicating on Survival Data According to the Audience: A Tutorial on Different Measures Illustrated With Population-Based Cancer Registry Data. Clin Epidemiol (2019) 11:53–65. doi: 10.2147/CLEP.S173523 30655705 PMC6322561

[26] Obrocea FL Sajin M Marinescu EC Stoica D. Colorectal Cancer and the 7th Revision of the TNM Staging System: Review of Changes and Suggestions for Uniform Pathologic Reporting. Rom J Morphol Embryol (2011) 52(2):537–44. 21655640

[27] van Steenbergen LN Elferink MAG Krijnen P Lemmens V Siesling S Rutten HJT. Improved Survival of Colon Cancer Due to Improved Treatment and Detection: A Nationwide Population-Based Study in The Netherlands 1989-2006. Ann Oncol (2010) 21(11):2206–12. doi: 10.1093/annonc/mdq227 20439339

[28] Feinstein AR Sosin DM Wells CK. The Will Rogers Phenomenon. Stage Migration and New Diagnostic Techniques as a Source of Misleading Statistics for Survival in Cancer. N Engl J Med (1985) 312(25):1604–8. doi: 10.1056/NEJM198506203122504 4000199

[29] Shahrier M Ahnen DJ. Colorectal Cancer Survival in Europe: The Will Rogers Phenomenon Revisited. Gut (2000) 47(4):463–4. doi: 10.1136/gut.47.4.463 PMC1728087 10986203

[30] McClements PL Madurasinghe V Thomson CS Fraser CG Carey FA Steele RJ. Impact of the UK Colorectal Cancer Screening Pilot Studies on Incidence, Stage Distribution and Mortality Trends. Cancer Epidemiol (2012) 36(4):e232–42. doi: 10.1016/j.canep.2012.02.006 22425027

[31] Levin TR Corley DA Jensen CD Schottinger JE Quinn VP Zauber AG. Effects of Organized Colorectal Cancer Screening on Cancer Incidence and Mortality in a Large Community-Based Population. Gastroenterology (2018) 155(5):1383–91.e5. doi: 10.1053/j.gastro.2018.07.017 30031768 PMC6240353

[32] Kubisch CH Crispin A Mansmann U Goke B Kolligs FT. Screening for Colorectal Cancer Is Associated With Lower Disease Stage: A Population-Based Study. Clin Gastroenterol Hepatol (2016) 14(11):1612–8.e3. doi: 10.1016/j.cgh.2016.04.008 27085763

[33] Mandel JS Church TR Bond JH Ederer F Geisser MS Mongin SJ. The Effect of Fecal Occult-Blood Screening on the Incidence of Colorectal Cancer. N Engl J Med (2000) 343(22):1603–7. doi: 10.1056/NEJM200011303432203 11096167

[34] Faivre-Finn C Bouvier-Benhamiche AM Phelip JM Manfredi S Dancourt V Faivre J. Colon Cancer in France: Evidence for Improvement in Management and Survival. Gut (2002) 51(1):60–4. doi: 10.1136/gut.51.1.60 PMC1773269 12077093

[35] Kim JH. Chemotherapy for Colorectal Cancer in the Elderly. World J Gastroenterol (2015) 21(17):5158–66. doi: 10.3748/wjg.v21.i17.5158 PMC4419056 25954089

[36] Colon Cancer Laparoscopic or Open Resection Study G Buunen M Veldkamp R Hop WC Kuhry E Jeekel J. Survival After Laparoscopic Surgery Versus Open Surgery for Colon Cancer: Long-Term Outcome of a Randomised Clinical Trial. Lancet Oncol (2009) 10(1):44–52. doi: 10.1016/S1470-2045(08)70310-3 19071061

[37] Deijen CL Vasmel JE de Lange-de Klerk ESM Cuesta MA Coene PLO Lange JF. Ten-Year Outcomes of a Randomised Trial of Laparoscopic Versus Open Surgery for Colon Cancer. Surg Endosc (2017) 31(6):2607–15. doi: 10.1007/s00464-016-5270-6 PMC5443846 27734203

[38] Leung KL Kwok SP Lam SC Lee JF Yiu RY Ng SS. Laparoscopic Resection of Rectosigmoid Carcinoma: Prospective Randomised Trial. Lancet (2004) 363(9416):1187–92. doi: 10.1016/S0140-6736(04)15947-3 15081650

[39] Kapiteijn E Marijnen CA Nagtegaal ID Putter H Steup WH Wiggers T. Preoperative Radiotherapy Combined With Total Mesorectal Excision for Resectable Rectal Cancer. N Engl J Med (2001) 345(9):638–46. doi: 10.1056/NEJMoa010580 11547717

[40] Meulenbeld HJ van Steenbergen LN Janssen-Heijnen ML Lemmens VE Creemers GJ. Significant Improvement in Survival of Patients Presenting With Metastatic Colon Cancer in the South of The Netherlands From 1990 to 2004. Ann Oncol (2008) 19(9):1600–4. doi: 10.1093/annonc/mdn176 18467312

