# Supplementary material for: Trends in Staging, Treatment, and Survival in Colorectal Cancer Between 1990 and 2014 in the Rotterdam Study
Source: Front Oncol. 2022 Feb 16;12:849951. doi: 10.3389/fonc.2022.849951 (PMC8889566; doi:10.3389/fonc.2022.849951)
Supplement: Supplementary file 1 [file DataSheet_1.docx]

**Supplementary Table 1** Summary of differences in the American Joint Committee on Cancer (AJCC) staging system for the 4^th^ to the 7^th^ edition.

|  | **Diagnosed 1990-2002** | | **Diagnosed 2003-2014** | |
| --- | --- | --- | --- | --- |
|  | **4^th^ edition^1^** | **5^th^ edition^1^** | **6^th^ edition^1^** | **7^th^ edition^2^** |
| **Stage I** | T1-2N0M0 | T1-2N0M0 | T1-2N0M0 | T1-2N0M0 |
| **Stage II** | T3-4N0M0 | T3-4N0M0 | - |  |
| **Stage IIa** | - | - | T3N0M0 | T3N0M0 |
| **Stage IIb** | - | - | T4N0M0 | T4aN0M0 |
| **Stage IIc** | - | - | - | T4bN0M0 |
| **Stage III** | Any T, N1-3,M0 | Any T, N1M0 | - | - |
| **Stage IIIa** | - | - | T1-2N1M0 | T1-2N1M0 T1N2aM0 |
| **Stage IIIb** | - | - | T3-4N1M0 | T3-4aN1M0  T2-3N2aM0  T1-2N2bM0 |
| **Stage IIIc** | - | - | Any T, N2M0 | T4aN2aM0  T3-4aN2bM0  T4bN1-2M0 |
| **Stage IV** | Any T, any N, M1 | Any T, any N, M1 | Any T, any N, M1 | - |
| **Stage IVa** | - | - | - | Any T, any N, M1a |
| **Stage IVb** | - | - | - | Any T, any N, M1b |

**1:** *T1 = tumor invades submucosa; T2 = tumor invades muscularis propria; T3 = tumor invades through the muscularis propria to the subserosa; T4 = tumor invades directly into other organs or structures and/or perforates visceral peritoneum. N1 = metastasis to 1 to 3 regional lymph nodes; N2 = metastasis to four or more regional lymph nodes; N3= lymph node involvement along the major vascular trunk. M0 = no distant metastasis; M1 = distant metastasis*

**2:** *T1 = tumor invades submucosa; T2 = tumor invades muscularis propria; T3 = tumor invades through muscularis propria into subserosa; T4a = tumor penetrates to the surface of the visceral peritoneum; T4b = tumor directly invades or is adherent to other organs or structures. N1a = metastasis in 1 regional lymph node; N1b = metastasis in 2-3 regional lymph nodes; N1c = tumor deposit in the subserosa, mesentery, or nonperitonealized pericolic or perirectal tissue without regional lymph node metastasis. N2 = metastasis in 4 or more regional lymph nodes; N2a = metastasis in 4-6 regional lymph nodes; N2b = metastasis in 7 or more regional lymph nodes*

**Supplementary Table 2** Mean and median overall survival in months in colon and rectal cancer subjects of all stages combined

| **Date of diagnosis** | **Mean survival** | **95% CI** | | **Median survival** | **95% CI** | |
| --- | --- | --- | --- | --- | --- | --- |
|  |  | Lower limit | Upper limit |  | Lower limit | Upper limit |
| **Colon** |  |  |  |  |  |  |
| Before January 1, 2003 | 79.5 | 63.9 | 95.0 | 44.3 | 26.7 | 61.9 |
| After January 1, 2003 | 74.4 | 62.0 | 86.7 | 45.2 | 26.1 | 64.3 |
| **Rectum** |  |  |  |  |  |  |
| Before January 1, 2003 | 79.0 | 56.6 | 101.6 | 41.0 | 26.8 | 55.2 |
| After January 1, 2003 | 88.4 | 69.3 | 107.5 | 55.4 | 24.0 | 86.7 |

**Supplementary Figure 1** Overview of in- and excluded colorectal cancer subjects

Colorectal cancer diagnosis, *n*=587

Prevalent cases *n*=81

Incident cases *n*=506

Non-pathology confirmed *n*=17

Synchronous CRC, *n*=10

Diagnosed at autopsy, *n*=3

Carcinoma in situ *n*=6

History of malignancy *n*=74

Rectal cancer *n*=124

Colon cancer *n*=272

Included CRC cases *n*=396

Incident cases with no prior cancer diagnosis *n*=432

**Supplementary Figure 2.** Kaplan Meier Estimate of survival per disease stage
Absolute overall survival per disease stage in colon cancer patients and rectal cancer patients

**
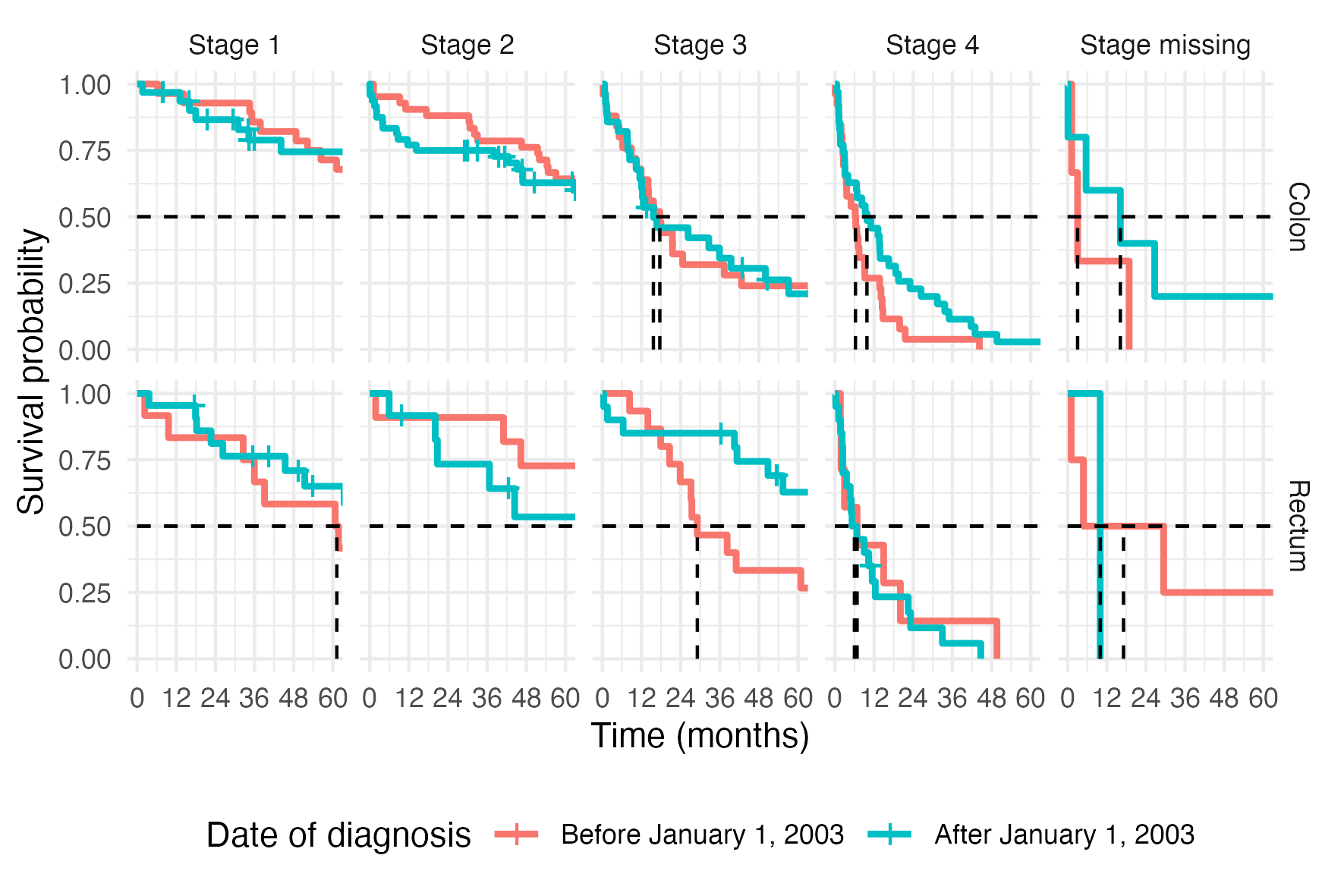
**

Legend: The 5-year overall survival (in months) in both colon cancer and rectal cancer per disease stage. The red line represents those diagnosed before January 1, 2003, the blue line those diagnosed after January 1, 2003.

**Supplementary Table 3.** Median overall survival in months per disease stage in colon cancer patients and rectal cancer patients

| **Disease stage** | **Colon cancer cohort** | | | | **Rectal cancer cohort** | | | |  |
| --- | --- | --- | --- | --- | --- | --- | --- | --- | --- |
|  | **1990-2003** | | **2003-2014** | | **1990-2003** | |  | **2003-2014** | |
|  | Median | 95% CI | Median | 95% CI | Median | 95% CI | | Median | 95% CI |
| I | 132.6 | 62.2-202.9 | 120.9 | 94.8-146.7 | 60.7 | 22.2–99.2 | | 118.9 | NA-NA |
| II | 85.9 | 57.0-114.9 | 82.6 | 75.3-89.9 | 120.9 | 70.1–171.7 | | 65.2 | 17.8–112.6 |
| III | 17.6 | 11.4-23.8 | 15.6 | 0.0-33.8 | 29.1 | 15.1–43.1 | | 90.9 | 9.3–172.6 |
| IV | 6.2 | 2.3-10.1 | 9.8 | 2.4-17.2 | 6.8 | 0.0–16.7 | | 5.2 | 1.6-8.9 |
| Missing | 3.1 | 0.2-5.9 | 16.2 | 0.0-38.7 | 4.8 | 0.0–32.6 | | 10.0 | NA-NA |
| Overall | 44.3 | 26.7-61.9 | 45.2 | 26.1-64.3 | 41.0 | 26.8–55.2 | | 55.4 | 24.0–86.7 |

NA = not applicable
CI = confidence interval

**Supplementary Table 4.** Cox Proportional Hazard model in colon cancer with outcome survival, adjusted for patient related factors, tumor characteristics and treatment.

| **Covariables** | | **HR** | **95% CI** | | **P-value** |
| --- | --- | --- | --- | --- | --- |
|  |  |  | **Lower limit** | **Upper limit** |  |
| Date of diagnosis | <01.01.2003 | Reference |  |  |  |
|  | >01.01.2003 | 1.28 | 0.84 | 1.95 | 0.25 |
| RS-cohort | I | Reference |  |  |  |
|  | II | 0.79 | 0.45 | 1.39 | 0.40 |
|  | III | 0.88 | 0.36 | 2.19 | 0.79 |
| Sex | Female | 0.57 | 0.371 | 0.878 | **0.01** |
| Age |  | 1.01 | 0.97 | 1.04 | 0.82 |
| BMI |  | 0.96 | 0.91 | 1.01 | 0.13 |
| Diabetes | Yes | 1.99 | 1.24 | 3.21 | **0.005** |
| Alcohol status | Never | Reference |  |  |  |
|  | Former | 0.98 | 0.40 | 2.44 | 0.97 |
|  | Current | 0.60 | 0.36 | 0.99 | **0.05** |
| Smoking | Never | Reference |  |  |  |
|  | Former | 1.57 | 1.01 | 2.42 | **0.04** |
|  | Current | 1.25 | 0.69 | 2.24 | 0.46 |
| SES | Very low | Reference |  |  |  |
|  | Low | 0.61 | 0.39 | 0.96 | **0.03** |
|  | Middle | 0.56 | 0.32 | 0.99 | **0.05** |
|  | High | 0.83 | 0.42 | 1.65 | 0.60 |
| Stage | I | Reference |  |  |  |
|  | II | 2.22 | 0.98 | 5.07 | 0.06 |
|  | III | 14.23 | 5.61 | 36.11 | **<0.001** |
|  | IV | 32.39 | 12.18 | 86.12 | **<0.001** |
| Morphology | Adenocarcinoma | Reference |  |  |  |
|  | Neuroendocrine | 1.28 | 0.15 | 10.97 | 0.82 |
|  | Squamous cell | 11.98 | 1.02 | 140.55 | **0.05** |
|  | Signet cell | 1.71 | 0.60 | 4.91 | 0.31 |
| Tumor site | Left-sided | Reference |  |  |  |
|  | Right-sided | 0.83 | 0.57 | 1.21 | 0.33 |
|  | Transverse colon | 1.54 | 0.75 | 3.19 | 0.24 |
| Relapse | None | Reference |  |  |  |
|  | Local | 0.77 | 0.18 | 3.29 | 0.73 |
|  | Local + distant | 0.55 | 0.12 | 2.51 | 0.44 |
|  | Only distant | 1.05 | 0.50 | 2.19 | 0.89 |
| Resection primary tumor | Yes | 0.72 | 0.33 | 1.55 | 0.40 |
| Open vs. laparoscopic | Open | Reference |  |  | **0.008** |
|  | Laparoscopic | 1.00 | 0.56 | 1.80 | 0.99 |
|  | Converted | 5.50 | 2.00 | 15.13 | **0.001** |
| Chemotherapy | None | Reference |  |  | **0.001** |
|  | Neoadjuvant | 0.19 | 0.02 | 2.25 | 0.19 |
|  | Adjuvant | 0.12 | 0.03 | 0.48 | **0.003** |
|  | Palliative | 0.15 | 0.06 | 0.36 | **0.001** |

**Supplementary Table 5.** Cox Proportional Hazard model in rectal cancer with outcome survival, adjusted for patient related factors, tumor characteristics and treatment.

| **Covariables** | | **HR** | **95% CI** | | **P-value** |
| --- | --- | --- | --- | --- | --- |
|  |  |  | **Lower limit** | **Upper limit** |  |
| Date of diagnosis | <01.01.2003 | Reference |  |  |  |
|  | >01.01.2003 | 0.31 | 0.13 | 0.74 | **0.008** |
| RS-cohort | I | Reference |  |  |  |
|  | II | 1.06 | 0.44 | 2.58 | 0.89 |
|  | III | 1.92 | 0.49 | 7.48 | 0.35 |
| Sex | Female | 0.50 | 0.23 | 1.08 | **0.08** |
| Age |  | 1.03 | 0.96 | 1.10 | 0.41 |
| BMI |  | 1.08 | 0.99 | 1.18 | 0.08 |
| Diabetes | Yes | 2.66 | 0.94 | 7.51 | **0.07** |
| Alcohol status | Never | Reference |  |  |  |
|  | Former | 4.73 | 0.77 | 29.19 | 0.31 |
|  | Current | 0.41 | 0.12 | 1.42 | 0.16 |
| Smoking | Never | Reference |  |  |  |
|  | Former | 1.06 | 0.38 | 2.96 | 0.92 |
|  | Current | 0.30 | 0.10 | 0.87 | **0.03** |
| SES | Very low | Reference |  |  |  |
|  | Low | 0.99 | 0.35 | 2.84 | 0.99 |
|  | Middle | 0.81 | 0.26 | 2.48 | 0.71 |
|  | High | 0.46 | 0.12 | 1.71 | 0.25 |
| Stage | I | Reference |  |  |  |
|  | II | 0.36 | 0.06 | 2.09 | 0.26 |
|  | III | 1.18 | 0.28 | 4.87 | 0.82 |
|  | IV | 22.26 | 4.27 | 116.16 | **<0.001** |
| Morphology | Adenocarcinoma | Reference |  |  |  |
|  | Signet cell | 3.58 | 0.10 | 134.82 | 0.49 |
| Relapse | None | Reference |  |  |  |
|  | Local + distant | 0.16 | 0.01 | 3.01 | 0.22 |
|  | Only distant | 0.70 | 0.13 | 3.69 | 0.67 |
| Resection primary tumor | Yes | 1.41 | 0.21 | 9.36 | 0.73 |
| Open vs. laparoscopic | Open | Reference |  |  |  |
|  | Laparoscopic | 1.67 | 0.60 | 4.63 | 0.32 |
|  | Converted | 8.86 | 1.21 | 64.80 | **0.03** |
| Chemotherapy | None | Reference |  |  |  |
|  | Neoadjuvant | 0.60 | 0.08 | 4.45 | 0.62 |
|  | Adjuvant | 0.39 | 0.04 | 4.04 | 0.43 |
|  | Palliative | 0.18 | 0.03 | 1.11 | **0.07** |
| Radiotherapy | None | Reference |  |  |  |
|  | Neoadjuvant | 1.68 | 0.45 | 6.20 | 0.44 |
